# Quantifying Rates of Evolutionary Adaptation in Response to Ocean Acidification

**DOI:** 10.1371/journal.pone.0022881

**Published:** 2011-08-09

**Authors:** Jennifer M. Sunday, Ryan N. Crim, Christopher D. G. Harley, Michael W. Hart

**Affiliations:** 1 Department of Biological Sciences, Simon Fraser University, Burnaby, British Columbia, Canada; 2 Department of Zoology, University of British Columbia, Vancouver, British Columbia, Canada; Biodiversity Insitute of Ontario - University of Guelph, Canada

## Abstract

The global acidification of the earth's oceans is predicted to impact biodiversity via physiological effects impacting growth, survival, reproduction, and immunology, leading to changes in species abundances and global distributions. However, the degree to which these changes will play out critically depends on the evolutionary rate at which populations will respond to natural selection imposed by ocean acidification, which remains largely unquantified. Here we measure the potential for an evolutionary response to ocean acidification in larval development rate in two coastal invertebrates using a full-factorial breeding design. We show that the sea urchin species *Strongylocentrotus franciscanus* has vastly greater levels of phenotypic and genetic variation for larval size in future CO_2_ conditions compared to the mussel species *Mytilus trossulus*. Using these measures we demonstrate that *S. franciscanus* may have faster evolutionary responses within 50 years of the onset of predicted year-2100 CO_2_ conditions despite having lower population turnover rates. Our comparisons suggest that information on genetic variation, phenotypic variation, and key demographic parameters, may lend valuable insight into relative evolutionary potentials across a large number of species.

## Introduction

The oceans have absorbed approximately 30% of anthropogenic CO_2_ emissions since the beginning of the industrial revolution [1], and the resulting increase in marine pCO_2_ has caused a decrease in both pH and the availability of free carbonate ions (CO_3_^2−^). By the end of this century, changes in carbonate chemistry of the open ocean's surface waters are expected to exceed those experienced within the past 20 million years [2], [3]. Despite the scale of change, the biological implications of this process, known as ocean acidification, are only beginning to be understood. Within-generation physiological responses have been documented in a wide range of marine species and across life-history stages, and include changes in rates of metabolism, growth, calcification, survival, and immune response (reviewed in [4], [5], [6]). At the population level, these changes are expected to affect species abundances [7], [8], [9], [10], [11] and global distributions [10].

Projections of future ecological change, however, hinge on the assumption that responses to elevated CO_2_ measured in present-day populations will apply to future populations, and by necessity overlook the potential for evolutionary change. Within-generation responses to global change may reflect the physiological acclimation capacity, or reaction norms, of individual organisms. However, cross-generational responses, and our understanding of the ecological impacts of climate change in general, depend critically on the extent to which populations will *evolve* in response to their changing environment [12], [13]. Intraspecific variation in response traits is ubiquitously apparent among individuals in ocean acidification impact studies, as evidenced by the reported error or standard deviation of traits, though the emphasis is usually on mean responses, and the variation among individuals is usually treated as noise [14]. While some of this ‘noise’ may be due to experimental error, a portion of this variation may represent genetic differences among individuals. The extent to which individual differences are genetically heritable within a population represents the potential for that population to adapt evolutionarily as a response to natural selection [14], [15], [16], [17].

Some key studies have revealed a potential for adaptation to elevated CO_2_. Artificial selection experiments have demonstrated evolutionary responses in a short-lived photosynthetic plant (changes in developmental rate) [18] and an aquatic alga (changes in photosynthesis and cell size) [19]. In the marine environment, comparisons among distinct clones of a coccolithophore species [20] and a bryozoan species [14] each demonstrated the existence a genetic basis for individual variation in fitness responses to pH. Moreover, artificial selection for fast growth and disease resistance in the aquaculture-bred Australian oyster *Saccostrea glomerata* resulted in selected lines that, serendipitously, suffered reduced effects of ocean acidification on shell growth compared to nonselected lines, indicating the existence of selectable genetic variation for CO_2_-dependent growth [21]. While these studies are valuable for understanding the potential for adaptation, most marine animals of ecological and economic interest have long generation times on the order of years to decades, are non-clonal, and have not previously been subjected to multiple generations of artificial selection, thus selection experiments and between-clone comparisons are limited approaches for characterizing the adaptive potential of species under ocean acidification.

In the present study, we use a full-factorial breeding design to estimate the additive genetic component of phenotypic variation in growth under present-day and near-future CO_2_ conditions within a single generation of two animals of ecological and economic importance. The bay mussel, *Mytilus trossulus*, directly provides complex habitat for other members of temperate intertidal communities [22], [23] and is part of a species complex used in a ca. $380M global aquaculture industry (Food and Agriculture Organization, 2010). The red sea urchin, *Strongylocentrotus franciscanus*, is a grazer of habitat-forming kelps [24], and the basis for a ca. $12M wild fishery in the Northeast Pacific Ocean (California and British Columbia combined; California Fish and Game, [25]. We focused on early larval development, a trait previously shown to respond strongly to ocean acidification in bivalves and echinoderms [26] and with important consequences through later stages of development [27], [28]. The aims of this study are threefold: for each species we (i) provide information on the effects of estimated year-2100 CO_2_ conditions on larval development (under the SRES A2 scenario [29]), (ii) estimate maternal and paternal sources of variation to calculate heritabilities and maternal effects under present and year-2100 CO_2_ conditions and (iii) simulate the response of each species to multiple generations of selection at year-2100 CO_2_ in order to compare the potentials for adaptive evolution in larval traits in either species.

## Methods

### Collection, transport, and husbandry

Male and female individuals were collected from sites in Barkeley Sound, British Columbia, and transported in vessels containing ambient seawater to the Bamfield Marine Sciences Center within 30 minutes of collection. Animals were maintained in large, covered tanks with flow-through, unfiltered seawater (11–13°C, pH unknown) for 1–2 weeks before use. *M. trossulus* were fed with organic particles in the seawater system, and *S. franciscanus* were fed with macrocystis kelp. Animals were kept and handled in accordance with the animal care guidelines of the center (Animal Use Protocol No. RS-09-13). See Methods S1 for details of collections sites.

### Spawning procedures

*M. trossulus* gametes were obtained from four females and ten males by warm-water spawning induction [30] or dissection (males only). Sperm were kept cold on ice in a minimal volume of filtered seawater. In *S. franciscanus*, gametes from 10 females and 10 males were obtained by spawning inductions using 0.5 M KCl injection through the peristome [30]. Sperm were collected ‘dry’ directly from the gonopores and kept cold in vials on ice until fertilization. For both species, eggs of each female were collected into 0.45 µm filtered seawater, and washed three times. Gametes were stored no longer than 2 hours prior to fertilization, and sperm were observed for motility before use.

### Fertilization cross

Male and female gametes of each species were combined in a factorial manner (North Carolina II cross; [31], [32]) to form 40 families of *M. trossulus* from 4 dams and 10 sires, and 100 families of *S. franciscanus* from 10 dams and 10 sires. In order to ensure that we sampled the effects of both paternal and maternal genetic variation on offspring phenotypes, we cultured larvae through the onset of obligate zygotic transcription in ambient seawater: for 24 hours in *M. trossulus* or 48 hours in *S. franciscanus* [33]. Larvae were then transferred to culture jars (22 ml glass vials) and brought to desired concentrations (3.3 larvae ml^−1^ for *M. trossulus*, and 1.8 larvae ml^−1^ for *S. franciscanus*) with treatment or control seawater (see below). All cultures for each experiment were kept in an incubator at 12°C with fluctuation below 1.5°C, and a 12 h day/night light cycle, and were rotated twice daily within the incubator.

### Seawater treatments

We manipulated CO_2_ in experimental seawater by mixing ambient air (400 ppm CO_2_) and CO_2_ gas (3% CO_2_, balance air; PraxAir) with Smart-Trak® mass flow controllers (Sierra Instruments, Inc.) to desired CO_2_ concentrations. Actual CO_2_ gas concentrations were verified with a Qubit S151 CO_2_ Analyzer. Experimental gases were vigorously bubbled into 20 L polyethylene carboys filled with 0.5 µm filtered seawater and allowed to equilibrate for at least 24 h. Carboys were kept at 11–13°C before use. We monitored the pH (NBS scale) of treatment and control water immediately before, during, and at the end of each experiment, using an Omega PHH-830 pH meter. For the *M. trossulus* experiment, high-CO_2_ water was equilibrated directly, using a gas mixture of 1000 ppm CO_2_, whereas for the *S. franciscanus* experiment, two batches of seawater that had been equilibrated with different CO_2_-enriched mixed air (800 and 1800 ppm) were combined equally to obtain water with approximately the same pH as that resulting from the 1000 ppm CO_2_ gas mixture (pH = 7.9). For both experiments, low CO_2_ treatments were obtained by running outdoor ambient air through the mass flow controllers and into a carboy to equilibrate with filtered seawater, in exactly the same way as the enriched gas (pH = 8.3). The resultant pH values, and differences between treatment and control, were consistent across species (Fig. S4). Total alkalinity was measured via Gran titration with an Accumet model 15 pH meter (Fisher Scientific). Salinity and total alkalinity were measured before each experiment, and did not differ between treatments or species (see Methods S1 for seawater chemistry details).

The procedures for maintaining pH differences for the two species differed as experimental techniques were developed. For *M. trossulus*, cultures were placed without lids in large airtight clear Plexiglas environmental control boxes, placed inside the incubation chamber. Gas inside these boxes was replaced with treatment or control mixed gas every 12 hours, to maintain CO_2_ concentrations. Each box contained one replicate of all 40 families in either a high or low CO_2_ treatment, and there were three replicates for each family and CO_2_ treatment (240 cultures, total). Extra cultures and seawater samples were placed in each box to monitor larval development and pH levels. The effect of common treatment box was minor and accounted for statistically in all analyses of CO_2_ and parental effects (see below and Methods S1).

*S. franciscanus* cultures were sealed in individual culture jars with a plastic screw-top lid, leaving a minimal (∼0.5 ml) ambient air bubble in each culture. Five replicate cultures of each CO_2_ treatment were made for all 100 families (1000 cultures, total). Extra cultures and seawater samples were made to monitor development and pH of treatments. For both *M. trossulus* and S. *franciscanus*, the effect of culture jars (either sealed with a lid or unsealed within an environmental control box) was accounted for statistically, thus accounting for differential variation introduced by this difference in experimental technique (see Methods S1 ‘Validating seawater treatment methods’ for more information).

### Measuring CO_2_ effects on development

**Figure 1:**
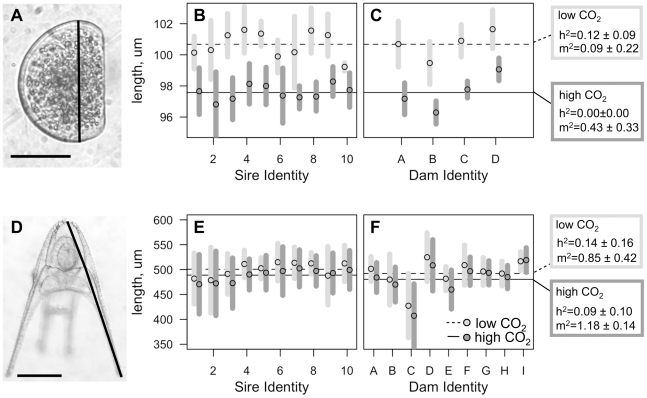
Variation in larval size under low and high CO_2_ conditions in *M. trossulus* and *S. franciscanus*. (**A**) *M. trossulus* larva at 65 h of development. (**B,C**) Variation in *M. trossulus* size at 65 hours in ambient CO_2_ (light grey) elevated CO_2_ (dark gray). (**D**) *S. franciscanus* larva at 7 days of development. (**E,F**) Variation in *S. franciscanus* size after 5 days in ambient CO_2_ (light grey) and elevated CO_2_ (dark gray). Vertical bars indicate ±1 standard deviation from the mean. The same data are shown partitioned among (**E**) sires and (**F**) dams. Horizontal lines represent means for ambient (dashed), and high (solid) CO_2_ treatments. Heritability calculated from sire-based additive genetic variance, and maternal effects indicating variance attributed to dams over-and-above sire-based heritability, are given for each treatment and each species (h^2^ = heritability, m^2^ = maternal effects). Lines on micrographs in (**A**) and (**D**) show linear measurements used to calculate rod lengths. Scale bars are 50 um in (**A**), and 150 um in (**D**).

We cultured larvae through to the end of their obligate nonfeeding period of morphogenesis, before the usual onset of feeding. We focused on heritability of growth variation in this early period of development in order to avoid potentially large artifacts of laboratory culture and feeding conditions on phenotypic variation in larval morphogenesis [30]. Larvae were fixed in 10% formalin buffered with CaCO_3_, photographed, and measured using ImageJ *1.41o* [34]. For *M. trossulus*, D-shaped larvae were fixed at 60 h of development, 6–10 larvae were sampled per vial (1741 individuals in total), and size was measured as the maximum anterior-posterior length parallel to the valve hinge (Fig. 1a). For *S. franciscanus*, four-armed echinopluteus larvae were fixed after 7 days of development, 8–10 larvae were sampled per vial (9989 individuals in total), and size was measured as post-oral arm length, body length, and body width. We used two metrics of size in *S. franciscanus*: overall larval length (sum of post-oral arm rod and body rod length, Fig. 1d) and the first principle component (PC1) of the these two metrics with length of the transverse body rod (Fig. S1). All subsequent results using either overall length or PC1 were quantitatively similar, so we show overall length results for easier comparison to mussel length metrics. Some preserved cultures of *S. franciscanus* lost skeletal arm rods during preservation, so only larvae with skeletal arm rods intact to effectively measure all 4 landmarks were used. Because larvae were not fed in culture, these linear dimensions of larvae (and differences among individuals and families) reflect differences in morphogenesis, but not an increase in total organic content.

Families of one *S. franciscanus* female were found to be highly affected by CO_2_ treatment, however this maternal family was also the first to be placed into control and treatment pCO_2_ conditions. As it was not possible to determine whether the strong effect of CO_2_ on this female was due to timing of treatment or to maternal effects specific to this female, all families with this female were discarded from further analyses.

### Quantifying CO_2_ effects

To determine the overall effect of CO_2_ and the contribution of parents to the overall variance in larval size, we fitted a mixed-effects linear model with CO_2_ treatment as a fixed effect, and sire, dam, sire*dam interaction, replicate culture, and treatment box (for *M. trossulus*), as crossed random effects, using the LME4 package in R [35], [36]. See Methods S1 for further details of linear models.

### Quantifying Heritability

Variance components of sire, dam, and sire*dam interaction were estimated separately for present-day and future CO_2_ conditions using ASReml [37]. For each treatment level, the following mixed-effects linear model was fit:
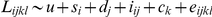
where *L_ijkl_* is the size of the *l*th sampled larva, within the *k*th incubation jar, from the *i*th dam and the *j*th sire; and sire (*s*), dam (*d*), sire-dam interaction (*i*), and culture (*c*) are random effects on the overall mean size (*u*). For *M. trossulus*, treatment box was also included as a random effect.

Following the sire model, we estimated sire-based additive genetic variance as four times the sire variance component, and phenotypic variance as the sum of sire, dam, sire*dam interaction, and residual variance components (hence culture and treatment box effects were excluded from phenotypic variance) [38]. Narrow sense heritability (hereafter, heritability or h^2^) was calculated as the ratio between sire-based genetic variation and total phenotypic variation.

The full-factorial cross also allows calculation of maternal effects (m^2^), as four times the difference between the dam and the sire variance components, divided by phenotypic variance [32]. A substantial effect of female parents over-and-above the effect of male parents is anticipated because the female contribution to offspring includes not only nuclear genes, but also all the components within an egg (maternal transcripts, organelles and nutrients), and may thus represent a combination of genetic and environmental variation. Because we cannot determine the proportion of maternal effects that are genetically based within a single-generation experiment, we allowed for the possibility that the maternal effects were anywhere from zero to 100% heritable in all subsequent simulations. Standard errors of heritability and maternal effects were calculated using the Delta method [32].

### Simulating evolutionary response to selection

To explore the potential implications of the genetic variation in development rate under high CO_2_, we used the breeder's equation [38] to predict the response to selection:
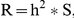
where S is the selection differential, or the difference between the population mean phenotype before and after selection; h^2^ is heritability; and R is the response to selection, or the change in the mean phenotype between generations.

To estimate the selection differential (S), we assumed that variation in larval size observed within treatments reflects variation in individuals' developmental rates, and is directly related to variation in larval duration required for individuals to become competent to metamorphose [39], [40], [41]. We then used published daily mortality rates based on *in situ* observations of bivalve and sea urchin larvae in the plankton to estimate a decline in fitness with longer larval durations [42]. We used the coefficient of phenotypic variation in size, observed for each species, and applied this to a mean expected larval duration (30 days for *M. trossulus* and 50 days for *S. franciscanus*, at 12°C) to obtain an expected mean and variation in larval duration for each species. Alternatively, there could be little or no change in the time to metamorphic competency, and the processes leading to smaller larval sizes in our experiments could result in smaller, less developed, or nutritionally compromised larvae at the time of metamorphosis [43]. Nevertheless, we can expect an associated fitness cost with each of these outcomes. For simplicity, we modeled the fitness function of development time because there are more empirical data on the costs of longer planktonic duration (see below), and less is known about the latent effects of larval size at metamorphosis (or other aspects of larval and juvenile quality) upon adult fitness [27].

We produced a fitness function for larval development time based on per-day mortality rates in the plankton due to predation, advection from suitable habitats, starvation, and disease [44]. Daily mortality rates from *in situ* observations have been estimated at 13–28% day^−1^ for bivalves and 6–26% day^−1^ for sea urchins [44]. To remain conservative, and because there is ultimately a limit to how quickly larvae can develop, we combined this decay function with an arbitrary decrease in fitness towards unrealistically short development times, such that a stable optimum would occur at intermediate larval durations, equal to the mean development time at ambient CO_2_ (30 days for *M. trossulus* and 50 days for *S. franciscanus*). The resultant fitness functions are shown in Fig. S2. Given these underlying functions, perturbations towards longer larval durations would result in decreased fitness.

We computed a selection differential (S) from the difference in mean development time before and after the fitness function was applied on a simulated population of larvae.

### Accounting for overlapping generations

In a species with overlapping generations, new recruits become sexually mature and join an existing population of surviving mature adults from previous years. To account for overlapping generations in our simulations, we included a population turnover rate, defined as the mean proportion of a breeding population made up of new recruits in a given year. Hence, the response to selection for each year was a weighted average of phenotypes from the existing population of surviving mature adults and the new recruits. To explore the sensitivity of model outcomes to different breeder turnover rates expected for each species, we allowed surviving larvae of *M. trossulus* to contribute 30, 50, or 90% of individuals to the next years' breeders, based on previous demographic observations in the focal region [45], and surviving larvae of *S. franciscanus* to contribute 10, 30, and 50% of individuals to the breeders 4 years post-metamorphosis, based on estimated demographic parameters [46].

To characterize the rate of adaptation, we iterated the breeder's equation with this breeder turnover rate for 50 years of evolution. This allowed 49 overlapping generations in *M. trossulus* (generation time of 1 year), and 45 overlapping generations in *S. franciscanus* (generation time of 5 years). We explored results using the range of heritabilities (h^2^) corresponding to maternal effects between zero and 100% heritable.

## Results

### Effect of CO_2_ on larval size

Atmospheric CO_2_ levels projected for year 2100 (under a SRES A2 scenario [29]) and associated decline of 0.31–0.33 pH units (Fig. S3) resulted in decreased size in both *Mytilus trossulus* and *Strongylocentrotus franciscanus* larvae (Fig. 1). The effect of elevated CO_2_ was similar between species: a 2.0% (*S. franciscanus*) to 3.2% (*M. trossulus*) decrease in larval body length (Fig. S4; Table S1). Including parental information in the random effects improved model fit, but was not required to detect a significant effect of the CO_2_ treatment (Table S1).

### Heritability, maternal effects, and scale of variation

For both species, sire-based heritabilities (h^2^) of larval size at age were low but non-zero under low pCO_2_, while at high pCO_2_ heritability was zero in *M. trossulus*, and low but non-zero in *S. franciscanus* (Fig. 1; Table S2). Maternal effects (m^2^) were high for both species under both conditions (Fig. 1; Table S2). Mothers producing large larvae did so at both low and high CO_2_ conditions, and quantitative estimates of maternal effects (m^2^) were similar under the two treatments (Fig. 1). Thus, the relative influence of individual dams on larval size was not influenced by CO_2_.

**Figure 2:**
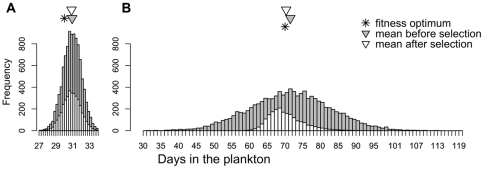
Phenotypic variation in planktonic duration at high CO_2_ before and after simulated selection for (A) *M. trossulus* and (B) *S. franciscanus*. Variation in planktonic duration was approximated from variation in size-at-day for either species under high CO_2_. Frequency of phenotypes before selection (dark bars) and after selection (light bars) are shown.

The amount of genetic and phenotypic variation contributing to these heritabilities greatly differed between sea urchins and mussels. Coefficients of phenotypic and additive genetic variation in size were 46 and 137 times greater in *S. franciscanus* than in *M. trossulus*, respectively (Table S2; Fig. 2). Because heritabilities are calculated from the ratio of additive genetic variation and phenotypic variation, we found similar heritabilities for both highly variable (sea urchin) and less variable (mussel) larval sizes.

### Simulated selection

After one generation of simulated selection on a cohort of larvae, the response to selection was much greater in *S. franciscanus* compared to *M. trossulus* - mean larval duration in *S. franciscanus* at high CO_2_ more nearly approached the mean larval duration at low CO_2_ (Fig. 2).

**Figure 3:**
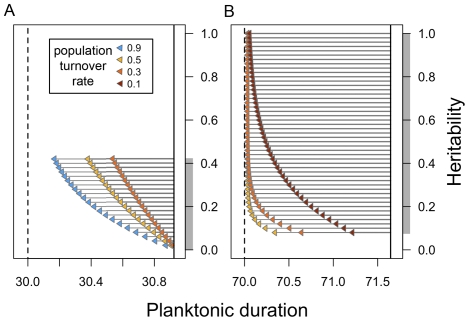
Summary of simulated evolution over 50 years, using different underlying heritabilities and population turnover rates. Solid vertical lines indicate the estimated mean planktonic duration at elevated CO_2_, and dashed vertical lines indicate mean planktonic duration at ambient CO_2_, or the ‘target’ of selection, for (**A**) *M. trossulus* and (**B**) *S. franciscanus*. Grey shading along the y-axis shows the range of possible heritabilities given maternal-effect heritability of 0 to 100%. Arrowheads indicate the mean phenotype after 50 years of evolution, and arrow lengths indicate the change in mean phenotype from the initial mean phenotype towards the target of selection. Population turnover rates used in simulations are shown in colour. Higher turnover rates are shown for *M. trossulus* (0.3–0.9) compared to *S. franciscanus* (0.1–0.5) to reflect known differences in species' demography (see methods).

When we simulated the effects of this difference over time by allowing post-selection cohorts of larvae to recruit into and mix with the surviving adult population, and iterated over 50 years of simulated selection, *S. franciscanus* had faster rates of simulated evolution than *M. trossulus*. This occurred over most of the range of possible maternal-effect heritabilities and population turnover rates (Fig. 3): under almost all combinations, sea urchin populations under high pCO_2_ reached the low-pCO_2_ phenotype within 50 years of selection (long grey lines spanning the range of planktonic durations in Fig. 3b), but no combination of parameters resulted in mussel populations reaching that same target of selection (shorter grey lines in Fig. 3a). As expected, greater population turnover rates lead to faster rates of phenotypic evolution. However the effect was not linear; the difference between a 10% and a 30% breeder replacement had a disproportionately large effect on the overall scope for adaptation in *S. franciscanus* than the difference between a 30% and 50% replacement rate (Fig. 3b).

## Discussion

This work represents the first quantitative assessment of evolutionary potentials in larval development under elevated CO_2_ to our knowledge. Our results support recent discoveries of genetically-based variation in CO_2_ responses of other life-history traits in other animals [14]. In both sea urchins and mussels, we find high maternal effects and low sire-based heritabilities for larval size-at-age in a year 2100-simulated CO_2_ environment. However, these species differ vastly in their coefficients of genetic and phenotypic variation, which leads directly to substantial differences in their simulated rates of evolution. We consider the role of maternal effects, phenotypic variation, demographic parameters, and the fitness associated with growth rates, for understanding the evolutionary responses of organisms to future ocean acidification.

For both species, the consistent strength and pattern of maternal differences in larval size at low and high CO_2_ indicates that egg provisioning does not interact with the effect of ocean acidification, but contributes additively to that effect. Hence, greater maternal provisioning may safeguard larvae in preparation for a high CO_2_ world, but cannot mitigate the effects entirely. A focus on the genetic or environmental factors that influence maternal egg provisioning may therefore help to identify those individuals or populations with stronger maternal buffering against the effects of ocean acidification. Maternal effects may themselves be heritable, as daughters may inherit their mothers' ability to provision eggs, and multigenerational studies are required to quantify the extent to which they are genetically heritable [32], [38], [47].

While the maternal effects tend to reduce the precision of heritability estimates and of the absolute rate of evolution, they do not greatly confound the comparison of relative rates of evolution between the two species. *Strongylocentrotus franciscanus* had a faster evolutionary rate compared to *Mytilus trossulus* over most of the parameter space for the heritability of maternal effects. This is because of the vastly different scales of phenotypic variation between the two species; greater phenotypic variation in *S. franciscanus* provides more variation on which selection can act, and leads to a greater differential in mean trait values before and after selection. Because the response to selection is a function of the selection differential *and* the heritability of a trait (according to the breeders' equation; *R = h^2^S*), two species with the same heritability can have different responses if one species has greater absolute amounts of phenotypic variation on which selection can act.

Evolution can occur more rapidly if more offspring surviving selection become incorporated into a population every year. In our simulations, population turnover rate had a direct effect on the rate of evolution, so uncertainty about the magnitude of this parameter in nature will lead to uncertainty in predictions about relative rates of evolution in natural populations. Population turnover rates are likely to differ greatly among marine species with vastly different reproductive modes, life histories, and demographic processes. In the northeast Pacific, individuals of *M. trossulus* tend to form ephemeral populations in disturbed habitat patches associated with its larger conspecific, *M. californianus*, and a large proportion of breeders are thought to be replaced every year [45]. In contrast, *S. franciscanus* have longer life-spans of many decades, with a much smaller proportion of adults replaced annually by new recruits [46]. Sea urchins nevertheless had faster evolutionary rates than mussels over most of the parameter space; only at the lowest population turnover rate for sea urchins (0.1) and the high rates in *M. trossulus* (0.9) did both species have a similar rate of evolution. Accurate estimation of breeder replacement is therefore an important aspect of predicting relative rates of evolution under climate change.

Another important parameter is the fitness function associated with variation in the traits affected by ocean acidification. We based our general fitness function on two assumptions: (i) that variation in size-at-day at high CO_2_ reflects variation in individual developmental rates, and (ii) that slower rates of development have a direct fitness cost due to greater mortality in the plankton. Support for the slower-rate assumption comes from accumulating observations of longer developmental times in planktotrophic larvae in acidified conditions [48], as well as direct observations of lower growth rates in *Mytilus edulis* [49]. A fitness cost associated with slower development is supported by direct observations of natural selection for larvae with faster development [39], [50] as well as evidence that pelagic durations are similar across climates [42], suggesting that molecular processes have evolved to be faster in colder climates to counter slower metabolism [42], [51]. Uncertainty in the shape of the fitness function leads to uncertainty in the absolute estimated rates of evolution, but this is not likely to affect the magnitude of difference between the two species, unless the shape of the fitness function is indeed different in either species. Nevertheless, future studies of evolutionary responses to CO_2_ will benefit from directly measuring fitness traits (e.g., effect of CO_2_ on survival or reproduction), or measuring entire larval durations [48], [49], [52].

Taken together, our comparisons suggest that knowledge of among-species differences in (i) the scale of phenotypic variation, (ii) population turnover rates, and (iii) the fitness cost associated with trait variation may be as important as knowledge of heritability itself in predicting the evolutionary responses to ocean acidification. A genetic basis for variation in CO_2_ responses has been found in the three previous studies in which it has been sought [14], [20], [21] supporting the notion that genetic variation exists at some level for almost all quantitative characters [53]. Using this assertion, Lynch and Lande [54] developed a framework for predicting the adaptive fates of populations under global change based on fitness functions and phenotypic variation alone. In our study, the difference between *S. franciscanus* and *M. trossulus* was apparent despite the imprecision of heritability estimates, because of the large differences in phenotypic variation. Given the need to anticipate the impacts of global change rapidly on a wide range of species, estimates of phenotypic variation, particularly of fitness traits, may prove valuable for identifying species with greater or less potential for adaptation. While estimates of genetic variation require complex mating or multiple-generational designs, we suggest that biologists report the inter-individual variation in ocean acidification impact studies, so that at least phenotypic variation can be estimated across a broad range of species.

We have demonstrated how variation in individual responses to ocean acidification can be used to understand species-specific differences in evolutionary potentials. The larvae generated for use in ocean acidification experiments lend themselves to such analyses, and require (minimally) that larvae of different parents be raised separately, that parental information be retained for every individual, and that adequate replication is made within families to estimate the environmental source of variation. Steps to reduce the signal of maternal effects will be valuable, such as quantification of egg traits prior to fertilization, and raising larvae for longer time periods to reduce the effects of variation in egg provisioning. Improved estimates of evolutionary rates will benefit not only from further empirical support for fitness functions and demographic population turnover rates, but also consideration of (i) the role of genetic correlations between responses to different variables (particularly with regards to pH and temperature) or between life stages [55], [56]; (ii) the potential for demographic change within species as a result of climate change, such as the rate of inbreeding [57], and (iii) the rate at which genetic variation can be maintained after several generations of directional selection [58]. Addressing these challenges will not be trivial, and will require concerted, cross-disciplinary work involving evolutionary biologists, oceanographers, physiologists and ecologists.

## Supporting Information

Methods S1 (DOC)

Table S1 **Effect of CO_2_ treatment on length variation in** ***M. trossulus*** **and** ***S. franciscanus*** **.** Akaike Information Criterion (AIC) comparison indicates that top models include maternal and paternal sources as random effects.
(DOCX)

Table S2 **Phenotypic variance, additive genetic variance, heritability and maternal effects for larval size in** ***M. trossulus*** **and** ***S. franciscanus*** **within CO_2_ treatments (± SEM).**
(DOCX)

Figure S1 **Variation in size-at-day under low and high CO_2_ conditions in** ***S. franciscanus*** **, using the first principle component (PC1) of three skeletal rod lengths.** (**A**) *S. franciscanus* larva at 7 days of development. Lines indicate length measurements for body length (*l*), post-oral arm (*a*), and transvers body rod (*t*) used in principle components analysis. Scale bar is 150 µm. (**B,C**) Variation in *S. franciscanus* PC1 after 5 days in ambient CO_2_ (light grey bars) and elevated CO_2_ (dark gray bars). Vertical bars indicate ±1 standard deviation from the mean. The same data are shown partitioned among (**B**) sires and (**C**) dams. Horizontal lines represent means for ambient (dashed), and high (solid) CO_2_ treatments. Heritability calculated from sire-based additive genetic variance, and maternal effects indicating variance attributed to dams over-and-above sire-based heritability, are given for each treatment (h^2^ = heritability, m^2^ = maternal effects). PC1 loadings were: a = 0.51, b = 0.58, c = 0.63, all loading with the same sign, and representing 0.53 of the variance.
(TIF)

Figure S2 **Hypothesized fitness functions for development time used in evolutionary simulations of (A)** ***Mytilus trossulus*** **and (B)** ***Strongylocentrotus franciscanus*** **.** Red lines represent the loss of fitness with greater development time due to a constant daily mortality rate in the plankton of 15% day^−1^, blue lines represent the loss of fitness expected with extremely rapid (short) development times, and black lines represent the resulting fitness function, the product of the red and blue fitness functions. The shape of the blue fitness function was arbitrarily chosen such that the equilibrium of the resultant fitness function (black) would center on the development time under low CO_2_ for each species.
(TIF)

Figure S3 **Separations in pH values between low (blue) and high (red) CO_2_ treatments during experiments, for (A)** ***M. trossulus*** **and (B)** ***S. franciscanus*** **.** Error bars indicate standard deviation. Although among-culture variance in pH was greater in the *S. franciscanus* experiment, variance in developmental responses attributed to culture jar was not included in calculation of genetic or phenotypic variance (culture effects were controlled for; see methods and Methods S1).
(TIF)

Figure S4 **Effect of CO_2_ treatment on** ***Mytilus trossulus*** **(A) and** ***Strongylocentrotus franciscanus*** **(B) larval size.** Mean size from each culture is shown in grey, and data aggregated by sire and dam combination (family mean) are shown in black. Segments connect family means at high and low CO_2_ treatments.
(TIF)
