# Supplementary material for: Quantifying Rates of Evolutionary Adaptation in Response to Ocean Acidification
Source: PLoS One. 2011 Aug 9;6(8):e22881. doi: 10.1371/journal.pone.0022881 (PMC3153472; doi:10.1371/journal.pone.0022881)
Supplement: Methods S1 — (DOC) [file pone.0022881.s001.doc]

**Supporting Methods S1**

**Collection sites**

Adult specimens of *Mytilus trossulus* were collected during low tide from semi-exposed intertidal shores of Dixon Island, in Imperial Eagle Channel, Bamfield, British Columbia. Adult specimens of *Strongylocentrotus franciscanus* were collected by SCUBA from two sites within Imperial Eagle Channel: Eagle Island and Wizard Island. At both sites, *S. franciscanus* were collected along rocky slopes between 15 and 20 m in depth. Collections and experiments were done in June, 2009.

**Seawater Chemistry**

Bubbling seawater with CO_2_-enriched air resulted in pH differences of -0.32 and -0.31 units from the control in the *M. trossulus* and *S. franciscanus* experiment, respectively. Salinity (mean ± st.dev. = 34.3±0.1 psu), temperature (12.03±0.27°C) and total alkalinity (2047.7±15.57 µmol kg^-1^ seawater) showed no differences across treatments (P > 0.21 in all cases, data not shown). We used these values to calculate pCO_2_ for each treatment, but the estimated pCO_2_ values were lower than expected for both treatments (low pCO_2_=253.5, high pCO_2_=570.8). We suspect that the cause of these anomalously low calculated pCO_2_ values was an error in either the pH meter (8.3 is a relatively high pH for ambient seawater), and/or the alkalinity estimate (expected alkalinity in this region given the temperature and salinity is about 2400 µmol kg^-1^, using methods in [1]. Nevertheless, the difference in pH values between treatments was similar to the expected difference between year 2010 and 2100 conditions [2].

**Validating seawater treatments during experiments**

Treatment differences in pH were similar between the two experiments (3.1 and 3.2 pH units; Fig. S3), though the pH in high CO_2_ treatments increased gradually over time in the *S. franciscanus* experiment. Among-culture variation in pH was also greater in the *S. franciscanus* experiment, particularly in the high CO_2_ treatment (Fig. S4), which was likely due to the different methods used to seal the culture containers (individually in the *S. franciscanus* experiment, as a group within boxes in the *M. trossulus* experiment). This was likely responsible for the greater among-culture variation in larval size in the high CO_2_ treatment in *S. franciscanus* (8.9% of variation attributed to culture effects in *S. franciscanus*, compared to 2.9% in *M. trossulus)*. In the *M. trossulus* experiment, there was an even larger effect of incubation box, which represented 15.0% of variation in larval length in the high CO_2_ treatment. However, the effect of CO_2_ and both phenotypic and genetic variation within CO_2_ treatments were calculated while controlling for the effect of culture (and for *M. trossulus* experiment, accounting for incubation box; see linear models in main text and below), such that phenotypic variance represents the sum of within-culture variance and variance attributed to parental sources, and explicitly *excludes* variance attributed to cultures and boxes.

**Quantifying effect of CO_2_: statistical details**

We fitted a mixed-effects linear model with CO_2_ treatment as a fixed effect, and sire, dam, sire*dam interaction, replicate culture, and treatment box (for *M. trossulus* only) as crossed random effects, using the LME4 package in R [3,4]. The significance of each effect was assessed by a log-likelihood ratio test between nested models with and without the random component of interest [5].

To test if accounting for parental source improved the power to detect a CO_2_ effect on larval size, we sequentially removed both dam and sire from the model and tested for an effect of CO_2_ on larval length. Models were compared using Akaike Information Criterion (AIC) [6].

# References

1. Lee K, Tong LT, Millero FJ, Sabine CL, Dickson AG, et al. (2006) Global relationships of total alkalinity with salinity and temperature in surface waters of the world's oceans. Geophysical Research Letters 33.

2. Nakicenovic N, Swart R (2000) Special Report on Emissions Scenarios. Cambridge, UK: Cambridge University Press.

3. Bates D, Maechler M (2009) lme4: Linear mixed-effects models using S4 classes. R package version 0.999375-32.

4. R Development Core Team (2009) R: A Language and Environment for Statistical Computing. Vienna, Austria.

5. Zuur AF, Ieno EN, Walker NJ, Savellev AA, Smith GM (2009) Mixed Effects Models and Extensions in Ecology with R. New York: Springer.

6. Burnham K, Anderson DR (1998) Model Selection and Inference: A Practical Information-Theoretic Approach. New York.: Springer-Verlag Telos.
