# Supplementary material for: Quantifying Rates of Evolutionary Adaptation in Response to Ocean Acidification
Source: PLoS One. 2011 Aug 9;6(8):e22881. doi: 10.1371/journal.pone.0022881 (PMC3153472; doi:10.1371/journal.pone.0022881)
Supplement: Table S1 — Effect of CO2 treatment on length variation in M. trossulus and S. franciscanus . Akaike Information Criterion (AIC) comparison indicates that top models include maternal and paternal sources as random effects. (DOCX) [file pone.0022881.s002.docx]

**Table S1.**  Effect of CO_2_ treatment on length variation in *M. trossulus* and *S. franciscanus*. Akaike Information Criterion (AIC) comparison indicates that top models include maternal and paternal sources as random effects.

| **Model statement** | |  | **Model AIC** | **Fixed effect** | | **Random effects** | | |  |  |  |  |  |  |  |
| --- | --- | --- | --- | --- | --- | --- | --- | --- | --- | --- | --- | --- | --- | --- | --- |
|  |  |  |  | CO_2_ treatment | | Dam |  | Sire |  | Dam*Sire | | Box |  | Culture | Resid. |
|  |  |  |  | contrast coef. (µm±SE) | p | vc | p | vc | p | vc | p | vc | p | vc | vc |
| *M. trossulus* | |  |  |  |  |  |  |  |  |  |  |  |  |  |  |
| L ~ | trmt + dam + sire + dam*sire + box + culture |  | **9287** | **3.10 ± 1.0** | 0.012 | **6.6** | <0.000 | 0.7 | 0.31 | 1.6 | 0.094 | **10.6** | <0.000 | **4.6** | 75.9 |
| L ~ | trmt + dam + box + culture |  | 9291 | **3.10 ± 1.0** | 0.012 | **6.6** | <0.000 |  | - |  | - | **10.6** | <0.000 | **6.7** | 76.2 |
| L ~ | trmt + box + culture |  | 9342 | **3.11 ± 1.0** | 0.009 |  | - |  | - |  | - | **10.3** | <0.000 | **12.6** | 77.1 |
|  |  |  |  |  |  |  |  |  |  |  |  |  |  |  |  |
| *S. franciscanus* | |  |  |  |  |  |  |  |  |  |  |  |  |  |  |
| L ~ | trmt + dam + sire + dam*sire + culture |  | **80195** | **11.15 ± 1.6** | <0.000 | **20.3** | <0.000 | 1.7 | 0.31 | **22.7** | <0.000 |  | - | **9.0** | 46.3 |
| L ~ | trmt + dam + culture |  | 80736 | **11.7 ± 2.5** | <0.000 | **22.4** | <0.000 |  | - |  | - |  | - | **31.8** | 45.8 |
| L ~ | trmt + culture |  | 81070 | **12.9 ± 3.1** | <0.000 |  | - |  | - |  | - |  | - | **53.7** | 46.3 |
| *Fixed effects*: CO_2_ treatment (trmt); *Random effects*: Dam, Sire, Dam and Sire interaction (Dam*Sire), treatment box (for *M. trossulus* only), and culture replicate. Bold indicates top AIC scores, and significant fixed and random effects. | | | | | | | | | | | | | | | |
