# Supplementary material for: Quantifying Rates of Evolutionary Adaptation in Response to Ocean Acidification
Source: PLoS One. 2011 Aug 9;6(8):e22881. doi: 10.1371/journal.pone.0022881 (PMC3153472; doi:10.1371/journal.pone.0022881)
Supplement: Table S2 — Phenotypic variance, additive genetic variance, heritability and maternal effects for larval size in M. trossulus and S. franciscanus within CO2 treatments (± SEM). (DOCX) [file pone.0022881.s003.docx]

**Table S2.** Phenotypic variance, additive genetic variance, heritability and maternal effects for larval size in *M. trossulus* and *S. franciscanus* within CO_2_ treatments (± SEM).

|  | | mean length | phenotypic variance | coefficient of phenotypic variance | sire-based additive genetic variance | coefficient of sire-based variance | dam-based additive genetic variance | coefficient of dam-based variance | heritability | maternal effects |
| --- | --- | --- | --- | --- | --- | --- | --- | --- | --- | --- |
|  |  |  |  |  |  | V_AS_/u |  | V_AD_/u | V_AS_ / V_P_ | (V_AS_-V_AD_)/ V_P_ |
| Species and treatment | | u | V_P_ | CV_P_ | V_AS_ | CV_AS_ | V_AD_ | CV_AD_ | h^2^ | m^2^ |
|  | | (µm) | (µm) |  | (µm) |  | (µm) |  |  |  |
| *M. trossulus* | |  |  |  |  |  |  |  |  |  |
|  | low CO_2_ | 100.6 ± 0.8 | 13.3 ± 1.0 | 0.13 | 1.6 ± 1.3 | 0.016 | 2.8 ± 2.6 | 0.028 | 0.12 ± 0.09 | 0.09 ± 0.22 |
|  | high CO_2_ | 97.6 ± 1.0 | 11.4 ± 1.2 | 0.12 | 0 ± 0 | 0 | 5.0 ± 4.2 | 0.051 | 0.00 ± 0.00 | 0.43 ± 0.33 |
| *S. franciscanus* | |  |  |  |  |  |  |  |  |  |
|  | low CO_2_ | 501.1 ± 9.2 | 2388 ± 401 | 4.77 | 341± 381 | 0.68 | 2380±1417 | 4.75 | 0.14 ± 0.16 | 0.85 ± 0.42 |
|  | high CO_2_ | 491.4 ± 10.5 | 2712 ± 492 | 5.52 | 248±285 | 0.51 | 3448 ± 1880 | 7.01 | 0.09 ± 0.10 | 1.18 ± 0.14 |
| Top row defines the variables shown in second row. | | | | | | | | | | |
