# Supplementary figures and images for: Quantifying Rates of Evolutionary Adaptation in Response to Ocean Acidification
Source: PLoS One. 2011 Aug 9;6(8):e22881. doi: 10.1371/journal.pone.0022881 (PMC3153472; doi:10.1371/journal.pone.0022881)

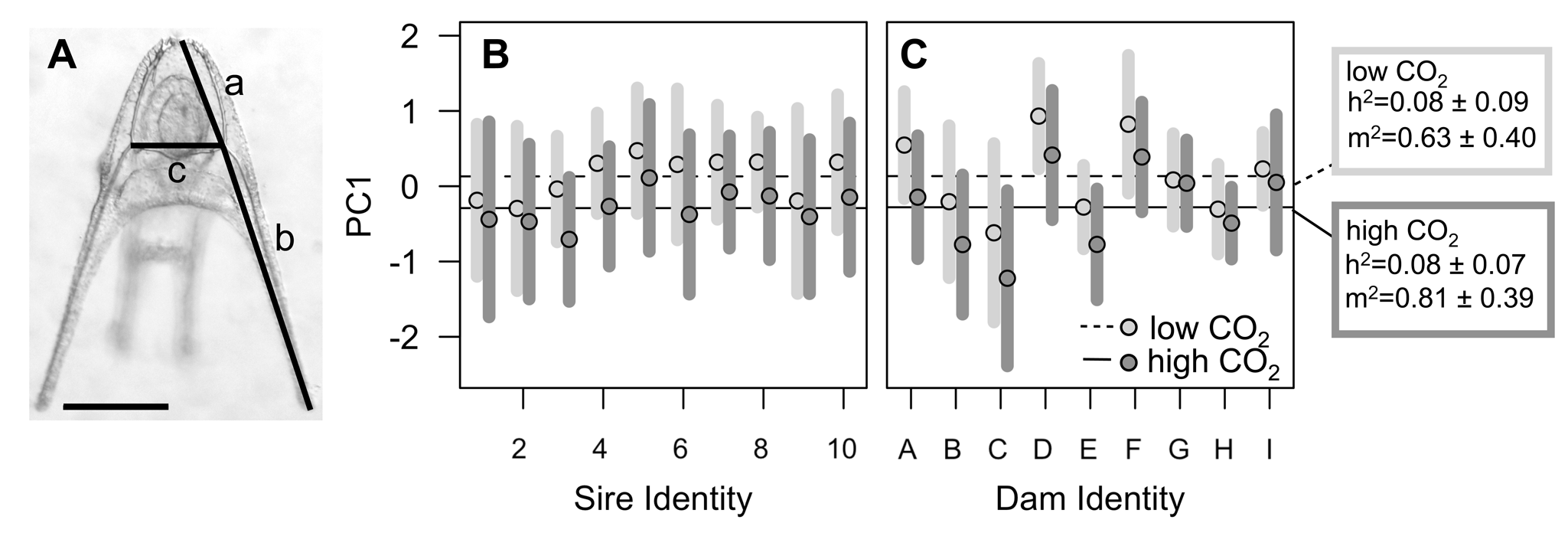

Supplement: Figure S1 — Variation in size-at-day under low and high CO2 conditions in S. franciscanus , using the first principle component (PC1) of three skeletal rod lengths. (A) S. franciscanus larva at 7 days of development. Lines indicate length measurements for body length (l), post-oral arm (a), and transvers body rod (t) used in principle components analysis. Scale bar is 150 µm. (B,C) Variation in S. franciscanus PC1 after 5 days in ambient CO2 (light grey bars) and elevated CO2 (dark gray bars). Vertical bars indicate ±1 standard deviation from the mean. The same data are shown partitioned among (B) sires and (C) dams. Horizontal lines represent means for ambient (dashed), and high (solid) CO2 treatments. Heritability calculated from sire-based additive genetic variance, and maternal effects indicating variance attributed to dams over-and-above sire-based heritability, are given for each treatment (h2 = heritability, m2 = maternal effects). PC1 loadings were: a = 0.51, b = 0.58, c = 0.63, all loading with the same sign, and representing 0.53 of the variance. (TIF) [file pone.0022881.s004.tif]

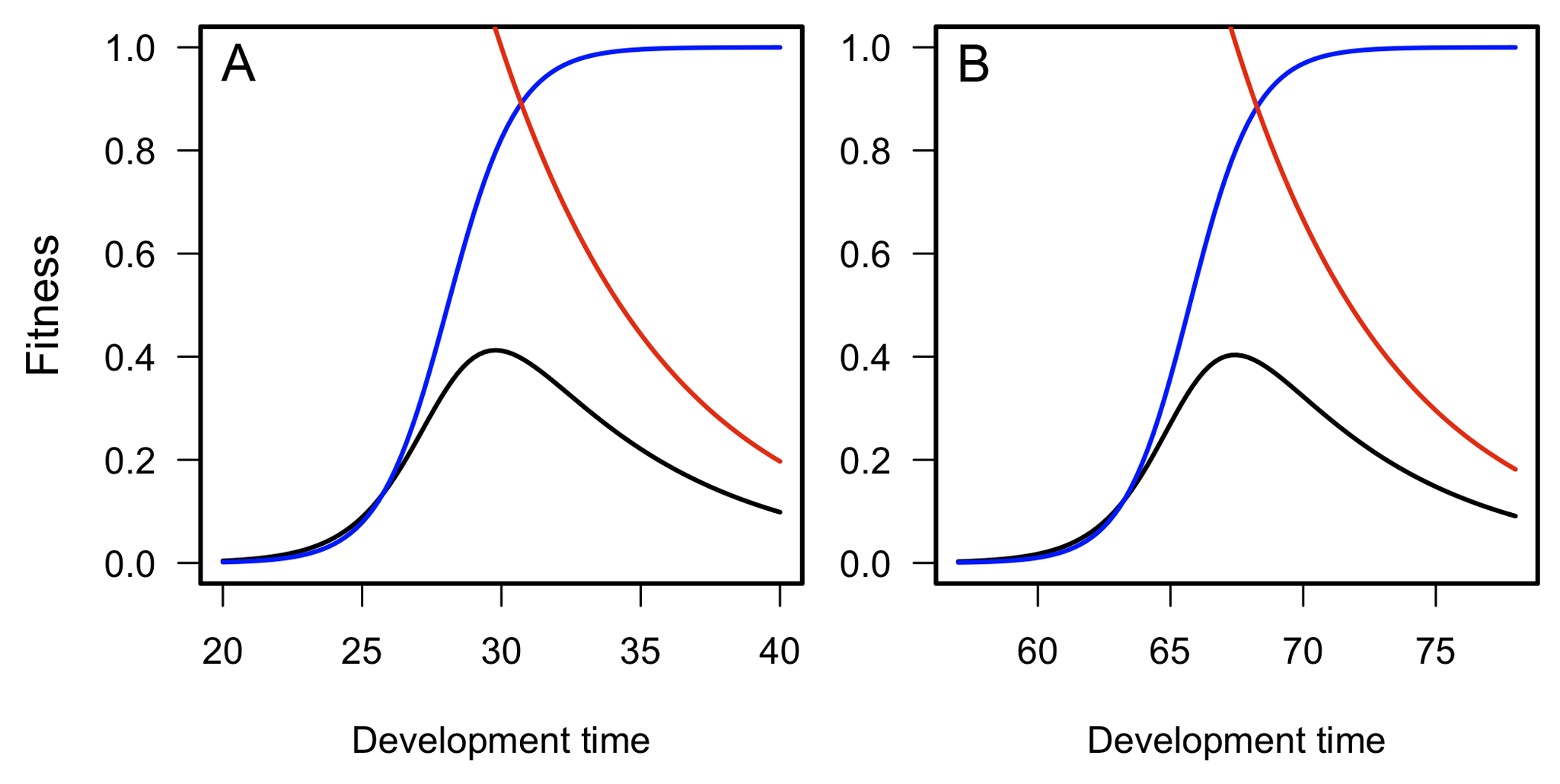

Supplement: Figure S2 — Hypothesized fitness functions for development time used in evolutionary simulations of (A) Mytilus trossulus and (B) Strongylocentrotus franciscanus . Red lines represent the loss of fitness with greater development time due to a constant daily mortality rate in the plankton of 15% day−1, blue lines represent the loss of fitness expected with extremely rapid (short) development times, and black lines represent the resulting fitness function, the product of the red and blue fitness functions. The shape of the blue fitness function was arbitrarily chosen such that the equilibrium of the resultant fitness function (black) would center on the development time under low CO2 for each species. (TIF) [file pone.0022881.s005.tif]

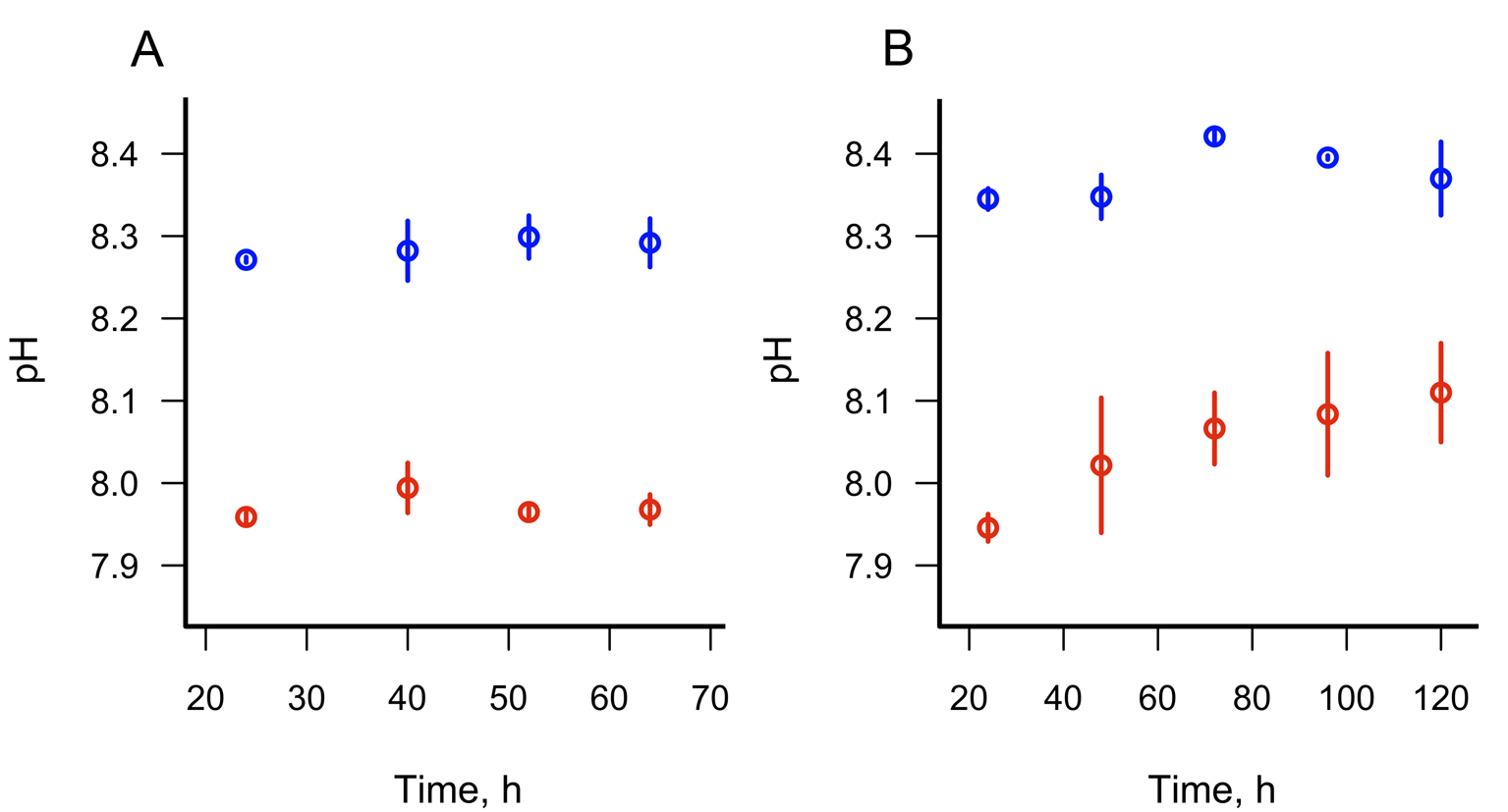

Supplement: Figure S3 — Separations in pH values between low (blue) and high (red) CO2 treatments during experiments, for (A) M. trossulus and (B) S. franciscanus . Error bars indicate standard deviation. Although among-culture variance in pH was greater in the S. franciscanus experiment, variance in developmental responses attributed to culture jar was not included in calculation of genetic or phenotypic variance (culture effects were controlled for; see methods and Methods S1). (TIF) [file pone.0022881.s006.tif]

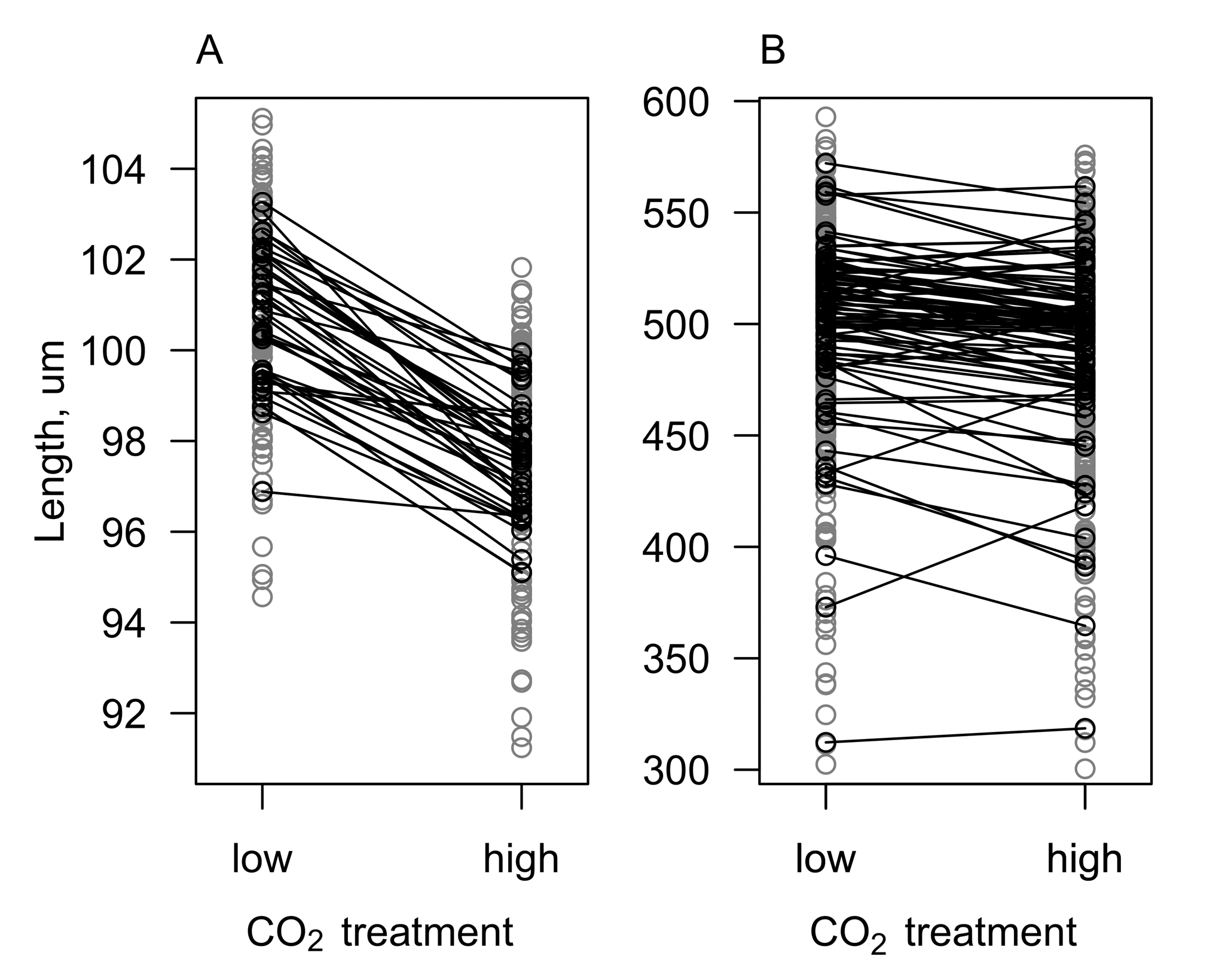

Supplement: Figure S4 — Effect of CO2 treatment on Mytilus trossulus (A) and Strongylocentrotus franciscanus (B) larval size. Mean size from each culture is shown in grey, and data aggregated by sire and dam combination (family mean) are shown in black. Segments connect family means at high and low CO2 treatments. (TIF) [file pone.0022881.s007.tif]
